# Expression Characteristics of Hypoxia-Inducible Factor-1α and Its Clinical Values in Diagnosis and Prognosis of Hepatocellular Carcinoma

**DOI:** 10.5812/kowsar.1735143X.771

**Published:** 2011-10-01

**Authors:** Shanshan Li, Dengfu Yao, Li Wang, Wei Wu, Liwei Qiu, Min Yao, Ninghua Yao, Haijian Zhang, Dandan Yu, Qichao Ni

**Affiliations:** 1 Research Center of Clinical Medicine, Affiliated Hospital of Nantong University, Nantong, China; 2 Department of Informatics, Affiliated Hospital of Nantong University, Nantong, China; 3 Department of Oncology, Affiliated Hospital of Nantong University, Nantong, China; 4 Department of General Surgery, Affiliated Hospital of Nantong University, Nantong, China

**Keywords:** Hypoxia-Inducible Factor 1, alpha Subunit, Carcinoma, Hepatocellular, Gene Expression, Vascular Endothelial Growth Factor, Angiopoietin-2

## Abstract

**Background:**

Hypoxia-inducible factor-1 (HIF-1) is a ubiquitously expressed oxygen-regulated transcription factor composed of α and β subunits. HIF-1 activates the transcription of various genes including those involved in the formation and metastatic growth of hepatocellular carcinoma (HCC).

**Objectives:**

To investigate the levels of hepatic and circulating HIF-1α expression in a range of patients with liver disease in order to determine how it can be used in the diagnosis of HCC and in establishing prognosis.

**Patients and Methods:**

Total RNA was extracted from a self-controlled HCC and paracancerous specimen. HIF-1α mRNA was amplified by nested RT-PCR and confirmed by sequencing. Tissue HIF-1α was analyzed by immunohistochemistry. The levels of HIF-1α, vascular endothelial growth factor (VEGF), and angiopoietin-2 (Ang-2) expression in the sera of 220 patients with liver disease were quantitatively detected by ELISA.

**Results:**

The positive staining of liver HIF-1α was brown and granule-like and was mainly present in the cytoplasm, with lower levels in the nucleus of hepatocytes. Its incidence was 80% in HCC cells and 100% in paracancerous tissues, with no significant difference in HIF-1α expression in relation to tumor number, degree of differentiation, or hepatitis B surface antigen (HBsAg) positivity, but with some correlation between HIF-1α and tumor size. HIF-1α expression was detected in the sera of HCC patients at a significantly higher level than in cases of benign liver disease, with pathological characteristics associated with the levels of circulating VEGF and Ang-2 expression, the size of the tumor, and the level of extrahepatic metastasis, but not with patients’ gender, age, or alpha-fetoprotein (AFP) levels.

**Conclusions:**

Hepatic HIF-1α expression is associated with the development and prognosis of HCC, and circulating HIF-1α level is a useful marker for HCC diagnosis and prognosis.

## 1. Background

Hepatocellular carcinoma (HCC) is one of the most common and rapidly fatal malignancies worldwide and has been ranked the second highest cancer killer in China since the 1990s, particularly in eastern and southern areas, including the inshore area of the Yangtze River [1]. Multiple risk factors are associated with HCC disease etiology, with the highest incidence in patients with chronic hepatitis B virus (HBV) and hepatitis C virus (HCV), although other factors such as genetic makeup and environmental exposure are involved [2][3][4]. As a common malignant solid tumor, HCC is characterized by fast infiltrating growth, early metastasis, high-grade malignancy, and poor therapeutic efficacy. It is a highly vascular tumor dependent on neovascularization and is one of the most common and rapidly developing malignancies [5][6]. HCC treatment options are severely limited by the frequent presence of metastases [7]. Multistep malignancy of HCC progression and multiple gene alterations are mostly accompanied by chronic hepatitis and liver cirrhosis [8][9].

Hypoxia-inducible factor-1 (HIF-1) is a basic helix–loop–helix Per–Arnt–Sim protein (bHLH-PAS) consisting of α and β subunits, and is a key transcription factor regulating cellular responses to hypoxia [10][11], and can regulate neovascularization and activate the expression of many hypoxia-response genes, leading to a close association with the HCC ecosystem for tumor growth, infiltration, metastasis, and prognosis [12][13]. HIF-1α is an oxygendependent protein, which is degraded by poly ubiquitination and proteasomal degradation via the Von Hippel– Lindau tumor suppressor protein under normoxic conditions [14][15][16]. Hepatic HIF-1α and its expression have been previously investigated during the malignant transformation of rat hepatocytes [17].

## 2. Objectives

In the present study, the expression and circulating levels of hepatic HIF-1α were investigated in patients with liver disease to prospectively elucidate the relationship between HIF-1α level and pathological characteristics, as well as the diagnosis and metastasis of HCC.

## 3. Patients and Methods

### 3.1. Patient Recruitment

A total of 131 HCC patients, 30 with chronic hepatitis, 22 with acute hepatitis, and 37 with cirrhosis (Table 1) were diagnosed at the Affiliated Hospital of Nantong University, Nantong, China, and samples from 27 healthy people were obtained from the Nantong Central Blood Bank as controls, with negative hepatitis viral markers (hepatitis B surface antigen [HBsAg], and anti-HCV antibody), normal alanine aminotransferase (ALT) levels, and Bultrasonic examination. All samples (5 mL of peripheral blood) were collected in the morning and sera were separated at once. Serum alpha-fetoprotein (AFP) concentrations exceeding 50 μg/L were taken as a positive result. The diagnosis of HCC and viral hepatitis was based on the criteria proposed by the Chinese National Collaborative Cancer Research Group [18] and at the Chinese National Viral Hepatitis Meeting [19], respectively.

**Table 1:** Patient Data in the Present Study

	**HCC a**	**LC a**	**CH a**	**AH a**	**NC a**
Patients, No.	131	37	30	22	27
Sex					
Male	109	16	25	17	12
Female	22	21	5	5	15
Age, y	33-85	20-82	18-63	24-80	26-69
HBsAg					
Positive	100	23	22	10	0
Negative	31	14	8	12	27
AFP, μg/L					
≤ 20	20	24	18	18	27
21-399	57	11	11	4	0
≥ 400	54	2	1	0	0

^a^ Abbreviations: AH, acute hepatitis; CH, chronic hepatitis; HCC, hepato- cellular carcinoma; LC, liver cirrhosis; NC, normal control

### 3.2. Liver Specimens

The self-controlled HCC and paracancerous specimens (2 cm to cancer) were collected from 35 patients who had undergone surgery for liver cancer at the Affiliated Hospital of Nantong University. The specimens were immediately frozen in liquid nitrogen and kept at -85°C until required. The patients included 28 men and 7 women, ranging in age from 22 to 70 years. Prior written informed consent was obtained from all patients according to the World Medical Association Declaration of Helsinki, and the study received ethics board approval from the Affiliated Hospital of Nantong University. The histologic types of all HCC specimens were graded in differentiation degrees as follows: good, 9; moderate, 12; and poor, 14. Of these specimens, 20 showed single tumor tubercles and the rest multiple; 14 were stage II, 13 were stage III, and 8 were stage IV. Each specimen was divided into 2 parts and analyzed by total RNA extraction and pathologic examination.

### 3.3. Total RNA Extraction

Total RNA was isolated from liver tissues by the guanidine thiocyanate method using the RNAzole reagent (Promega, Madison, WI) and purified as described elsewhere. The RNA was dissolved in tromethamine hydrochloride buffer (10 mmol/L, pH 8.0) containing EDTA, 10 mmol/L. The concentration of total RNA was assessed by optical density measurements at 260 nm in a UV spectrophotometer and expressed as total RNA micrograms per milligram of wet tissue and was then stored at -85°C.

### 3.4. Synthesis of Complementary DNA (cDNA)

For synthesis of cDNA, the reaction took place in a 20 μL reaction volume and was performed in a TC412 DNA thermal cycler (Techne, UK). Four micrograms of total RNA was denatured first in the presence of 1 μL random hexamers (Oligo-dT18, 0.5 μg/μL) with the addition of DEPC H2O to 12 μL, then in the presence of 4 μL 5× buffer, 1 μL RiboLockTM RNAase inhibitor (20 U/μL), and 2 μL dNTP mix (10 mmol/L), and finally with 1 μL RevertAidTM M-MuLV reverse- transcriptase (200 U/μL), at 70°C for 5 min, 37°C for 5 min, 42°C for 60 min, and 70°C for 10 min, and stored at -20°C for PCR amplification.

### 3.5. Amplification of Nested RT-PCR

The primers were designed using Premier Primer 5.0 Software based on the human HIF-1α sequence (NM_001530). The primers were synthesized using a synthesizer (Invitrogen, USA). The sequences of the 2 external primer pairs used for the initial PCR amplification were HIF-1α-P1 (sense): 5′-ATACTCAAAG TCGGACAGC-3′ (nucleotide [nt] 2386-2404) and HIF-1α-P2 (antisense): 5′-TT CACCCTGCAGTAGGTTTC-3′ (nt 2833-2852), and the size of the amplified gene fragment was 467 bp. The sequences of the 2 internal primer pairs used for the second PCR amplification were HIF-1α-P3 (sense): 5′-CTCATCCAAGAAGCCCTAAC- 3′ (nt 2452-2471) and HIF-1α-P4 (antisense): 5′-TCATAACTGGTCAGCTGTGG-3′ (nt 2781- 2800). The final size of the amplified gene fragment was 349 bp. PCR amplification consisted of initial denaturation at 94°C for 5 min, followed by 94°C for 25 s, 55°C for 30 s, and 72°C for 90 s for 30 cycles. The PCR products were electrophoresed on 2% agarose gels with ethidium bromide staining. The fragment sizes were evaluated using PCR markers (Promega) as molecular weight standards.

### 3.6. Sequencing of PCR Products

The 349-bp amplified product of the human HIF-1α gene was purified using a Montage PCR centrifugal filter device (Millipore, Billerica, MA) according to the manufacturer’s instructions. One microgram of DNA was used for preparation of the sequencing reaction and was directly sequenced using the MegaBACE DNA analysis system (MegaBACE DNA sequencer with the DYEnamic ET Dye Terminator Cycle Sequencing Kit; Amersham Biosciences, Piscataway, NJ), following the manufacturer’s protocol. The sequences were edited using the MegaBACE Sequence Analyzer, version 3.0 program (Amersham Biosciences) and the published HIF-1α gene was aligned with the amplified sequences from our human HCC and related paracancerous tissue samples.

### 3.7. Quantitative Detection of HIF-1α, VEGF and Ang-2 Levels

The levels of serum HIF-1α and VEGF (R&D systems, Abingdon, UK), and Ang-2 (ADL Biotech Dev Co., USA) were detected by ELISA in accordance with the manufacturer’s instructions. During the procedure, the plate was washed according to the routine ELISA method. Concentrations were calculated using a standard curve generated with specific standards provided by the manufacturer. Inter and intra-assay variations were lower than 10%.

### 3.8. Immunohistochemistry

The Polymer Detection System streptavidin-peroxidase (S-P) kit and positive control were purchased from Zhongshan Biotechnology Development Company, China. Serial 4-μm–thick paraffin sections were sequentially treated before application of primary antibodies in the following ways: deparaffinization, dehydration, endogenous peroxidase quenching, and antigen retrieval. Antigen retrieval for HIF-1α involved incubation with EDTA buffer in an autoclave. The sections were incubated with the monoclonal HIF-1α antibody (NeoMarkers, UK) at room temperature for 1 h. After the sections were washed with phosphate-buffered saline (PBS), the second antibody was added and incubated at room temperature for 30 min. The slides were then rinsed and the antibodies were detected by applying 3,3′-diaminobenzidine (DAB) as the chromogen for 5 min and a negative control with PBS was used substitute for the primary antibody. Breast cancerous tissue was used as an HIF-1α positive control. Expression of HIF-1α in 5 randomly selected microscopic fields (×200) was semi-quantitatively evaluated on the basis of the percentage of positive cells and classified as follows: when positive cells accounted for less than 10% of total cells, negative staining (-); 10–25%, weak staining (+); 26–50%, moderate staining (++); and > 51%, strong staining (+++).

### 3.9. Statistical Analysis

The patients were divided by diagnosis into HCC, acute hepatitis, chronic hepatitis, and liver cirrhosis, and healthy people served as the controls. Results are expressed as mean ± standard deviation (SD). Differences between groups were assessed by using a Student’s t test or a X(2) test. A P value of 0.05 or less was considered significant. Sensitivity and specificity were calculated according to the following formulas: sensitivity = a/ (a + c); and specificity = d/ (b + d), where a = true-positive cases, b = false-positive cases, c = false-negative cases, and d = truenegative cases. Receiver operating characteristic (ROC) curves [20] were constructed by calculating the sensitivities and specificities at several cutoff points.

## 4. Results

### 4.1. Expression of Hepatic HIF-1α in Liver Tissue

**Figure 1:**
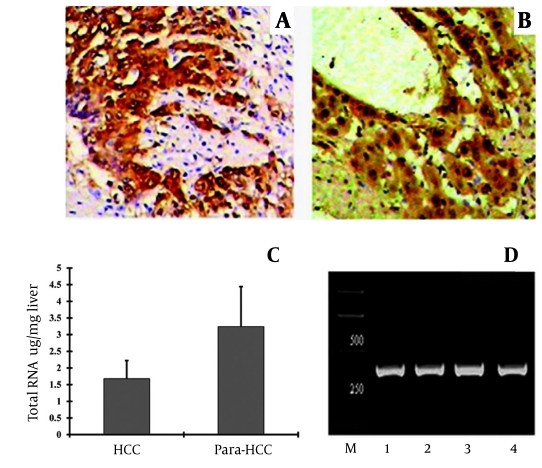
Immunohistochemical Staining of HIF-1α and Total RNA Levels and Amplification of HIF-1α mRNA in HCC or Associated Paracancerous Tissues. Hepatic HIF-1α expression can be visualized as brown particles in the cytoplasm and cell membrane. A, HCC tissue; B, paracancerous tissue (S-P, original magnification × 200); C, levels of total RNA expression in HCC or associated paracancerous tissues; D, HIF-1α mRNA was synthesized to HIF-1α cDNA and amplified by nested PCR (349 bp); Lines 1 and 2, amplified fragment of HIF-1α mRNA in HCC tissue; Lines 3 and 4, amplified fragment of HIF-1α mRNA in paracancerous tissue; M, DNA marker with molecular weight standard.HCC, hepatocellular carcinoma; Para-HCC, paracancerous tissues.

The expression and cellular distribution of HIF-1α in HCC tissues and comparative analysis with associated paracancerous tissues are shown in Figure 1. The positive staining of liver HIF-1α was brown and granule-like and mainly present in the cytosol, with lower levels in the nucleus. Positive cells were well distributed and in significant numbers in adjacent areas of necrosis and tumor infiltration in HCC (Figure 1A), whereas significant levels could be seen in compressed hepatic cords and the borders of central veins in the paracancerous tissues Figure 1B. Moreover, the HIF-1α positive staining was significantly higher (P = 0.017) in the paracancerous group (100%, 35 of 35) than in the corresponding HCC group (80%, 28 of 35). The intensity of hepatic HIF-1α expression was also higher in the paracancerous tissues than in the HCC tissues (Z = 4.728, P < 0.001, Table 2).

**Table 2:** Comparative Analysis of HIF a-1^a^ Expression Intensity in HCC or Associated Paracancerous Tissues (n = 35)

	**HCC a, No. (%)**	**Para-HCC, No. (%)**	***P *value b**	***Z *value**
HIF-1α positive	28 (80.0)	35 (100)	0.017	0.000
HIF-1α Intensity			0.000	4.728
-	7 (20)	0 (0)		
+	21 (60)	10 (28.57)		
++	7 (20)	18 (51.43)		
+++	0 (0)	7 (20)		

^a^ Abbreviations: HCC, hepatocellular carcinoma; HIF, hypoxia-inducible factor-1α

^b^ P value vs. the paracancerous tissue group

### 4.2. Expression of Total RNA and Amplification Analysis

**Figure 2:**
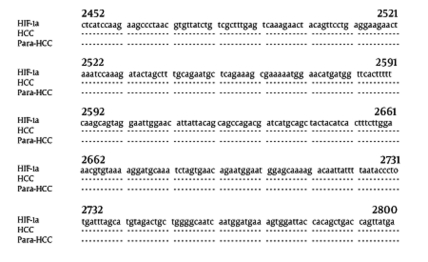
Alignment of the Amplified Fragments of the HIF-1α Gene and Homology Analysis of Their Sequences. HIF-1α: the cited sequence (349 bp, nt 2452-2800) of the human HIF-1α gene (NM_001530); HCC, amplified fragment of HIF-1α gene from HCC tissue; ParaHCC, amplified fragment of HIF-1α gene from associated paracancerous tissues.

Hepatic total RNA was purified from human HCC or associated paracancerous tissues. The specific concentrations of total RNA were 12.4 ± 7.3 μg/mg wet liver in the HCC group, and 53.8 ± 52.0 μg/mg wet liver in the paracancerous group (Figure 1C), with significant difference between them (t = 3.05, P < 0.01). The final amplified fragment of the hepatic HIF-1α gene was 349 bp (Figure 1D), and the incidence was 85.7% in the HCC group and 100% in the paracancerous group (P > 0.05). The amplified fragments of HIF-1α gene were confirmed by sequencing, and were completely consistent with the cited sequence of the human HIF-1α gene (Figure 2)

### 4.3. Expression of Circulating HIF-1α in HCC

**Figure 3:**
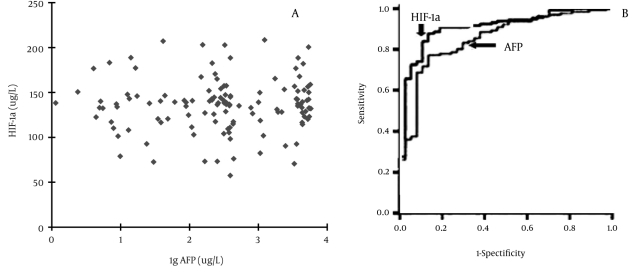
The Relationship between Circulating HIF-1α and AFP Level and Receiver Operating Characteristic (ROC) Curves (27). A, scatter diagram of circulating HIF-1α and AFP expression level in patients with HCC; B, ROC curves for circulating HIF-1α and AFP investigated markers for HCC. Sensitivity = true-positive rate; specificity = false-positive rate; and the area under the ROC curves was 0.854 for AFP and 0.909 for HIF-1α

The levels of circulating HIF-1α expression in 220 patients with liver disease are shown in Table 3. The circulating HIF-1α level was increased, particularly in patients with chronic liver disease. If the cutoff value of serum HIF-1α level was > 50 μg/L, the incidence of HIF-1α abnormality was 100% in HCC, 89.2% in liver cirrhosis, 66.7% in chronic hepatitis, and none in acute hepatitis or normal control groups. At a cutoff value of 100 μg/L, the abnormality of circulating HIF-1α level was 90.8% in HCC and 27.0% in liver cirrhosis, and none in chronic hepatitis, acute hepatitis, or normal control groups. The level of serum HIF-1α in HCC patients was significantly higher (P < 0.001) than that in cases with benign liver disease. The evaluation of serum HIF-1α and AFP levels for HCC diagnosis using ROC curves is shown in Figure 3. The advantage of analyzing 2 markers over the whole range of sensitivities and specificities using the area (0.854 in AFP, 0.909 in HIF-1α) under ROC curves indicated that the abnormality of serum HIF-1α level could be a useful serological marker for HCC diagnosis.

**Table 3:** Quantitative Analysis of Circulating HIF-1α Level (Mean ± SD) in Patients with Liver Disease

	**Patients, No. **	**HIF-1α a, μg/L**		**Cutoff Value**	
		**Range**	**Mean ± SD**	**> 50 μg/L, No. (%)**	**> 100 μg/L, No. (%)**
HCC a	131	57.5– 208.5	136.3 ± 28.8	131 (100)	119 (90.8)
LC a	37	39.1–123.4	84.6 ± 25.9 b	33 (89.2)	10 (27.0) b
CH a	30	38.0– 96.4	58.8 ± 14.5 c	20 (66.7) b	0 (0) b
AH a	22	33.1– 48.8	37.6 ± 5.3 d, e	0 (0) b	0 (0) b
NC a	27	20.3– 31.9	24.1 ± 3.3 f, g	0 (0) b	0 (0) b

^a^ Abbreviations: AH, acute hepatitis; CH, chronic hepatitis; HCC, hepatocellular carcinoma; HIF-1α, hypoxia-inducible factor-1α; LC, liver cirrhosis; NC, normal control

^b^ P < 0.001 vs. the HCC group

^c^ P < 0.001 vs. the liver cirrhosis group (q = 4.39)

^d^ P < 0.01 vs. the chronic hepatitis group (q = 3.17)

^e^ P < 0.001 vs. the liver cirrhosis group (q = 7.31)

^f^ P < 0.001 vs. the chronic hepatitis group (q = 5.47)

^g^ P < 0.001 vs. the liver cirrhosis group (q = 9.99)

### 4.4. Expression of Circulating VEGF and Ang-2 in HCC

The levels of circulating VEGF and Ang-2 expression in patients with chronic liver disease are shown in Table 4. As with circulating HIF-1α expression, the circulating VEGF and Ang-2 levels were increased in patients with chronic liver disease, particularly in HCC patients. If the cutoff value is > 280 μg/L for VEGF and > 35 μg/L for Ang-2, the incidence of VEGF and Ang-2 abnormality were 87.0% and 94.7% in HCC, 16.2% and 2.7% in liver cirrhosis, 13.3% and 0% in chronic hepatitis, and none in the normal control, respectively.

**Table 4:** Levels of Circulating VEGF a and ANG-2 a Expression in Patients with Chronic Liver Disease

	**Patients, No.**	**VEGF a, μg/L, Mean ± SD**	**> 280 μg/L, No (%)**	**ANG-2 a, μg/L, Mean ± SD**	**> 35 μg/L, No (%)**
HCC a	131	462.7 ± 119.2	114 (87.0)	40.8 ± 3.5	124 (94.7)
LC a	37	216.3 ± 54.5 b	6 (16.2) a	25.5 ± 5.8 b	1 (2.7) b
CH a	30	160.9 ± 98.2 b	4 (13.3) a	20.9 ± 7.1 b	0 (0) b
NC a	27	140.9 ± 54.5 b	0 (0) a	17.4 ± 2.6 b	0 (0) b

^a^ Abbreviations: ANG-2, angiopoietin-2; CH, chronic hepatitis; HCC, hepatocellular carcinoma; LC, liver cirrhosis; NC, normal control; VEGF, vascular endothelial growth factor

^b^ P < 0.001, vs. the HCC group

### 4.5. Clinicopathological Features of HIF-1α Expression

The expression of HIF-1α in HCC tissues correlated closely with the size of tumors; the incidence was 100% (14 of 14) in HCC tissues in the group with samples of more than 5 cm diameter, and 66.7% (14 of 21) in the group with samples of less than 5cm diameter (P = 0.017). No significant difference was found between hepatic positive HIF-1α expression and differentiation degree of tumor, tumor number, or HBsAg positivity (P > 0.05). The clinicopathological characteristics of circulating HIF-1α expression in HCC patients are shown in Table 5. Significant differences were found for high HIF-1α expression in relation to tumor size (P = 0.007) and HCC with extrahepatic metastasis (P < 0.001), but not in relation to patients’ gender, age, or AFP level (P > 0.05). There was a very close relationship between circulating HIF-1α level and VEGF (r = 0.937, P < 0.001) and Ang-2 (r = 0.933, P < 0.001), suggesting that high expression of HIF- 1α is associated with HCC metastasis and poor prognosis.

**Table 5:** Pathological Characteristics of HIF-1α Levels (mean ± SD) in Sera of HCC Patients

	**Patients, No. **	**HIF-1α a, μg/L**	*t ***value**	*P ***value**
HCC a	131	136.3 ± 28.8		
Sex			1.009	0.315
Male	109	137.4 ± 28.7		
Female	22	130.7 ± 29.0		
Age			1.702	0.091
≥ 50y	98	133.7 ± 30.1		
< 50y	33	143.2 ± 24.0		
Tumor size			2.721	0.007
≥ 5.0 cm	53	144.4 ± 26.3 b		
< 5.0 cm	78	130.8 ± 29.2		
AFP, μg/L			0.201	0.841
≥ 400.0	53	136.9 ± 25.8		
< 400.0	78	135.9 ± 30.7		
HBsAg			1.712	0.089
Positive	100	137.9 ± 29.6		
Negative	31	129.2 ± 25.5		
EHT a			5.522	0.000
Yes	49	152.5 ± 21.5 c		
No	82	126.6 ± 28.3		

^a^ Abbreviations: EHT, extrahepatic metastasis; HCC, hepatocellular carcinoma; HIF-1α, hypoxia-inducible factor-1α

^b^ P < 0.01 vs. the tumor size less than 5 cm group

^c^ P < 0.001 vs. the non-extrahepatic metastasis group

## 5. Discussion

HCC is one of the most common malignant cancers worldwide. Multiple risk factors are associated with HCC disease etiology, with the highest incidence in patients with chronic HBV or HCV infection, although other factors such as chemical carcinogenesis, alcohol consumption, exposure to dietary aflatoxin B1, activation of oncogenes, and inactivation of tumor suppressor genes are also involved [20][21]. HCC is known to contain aberrantly vascularized regions characterized by severe hypoxia [22]. Hypoxia can stimulate cell proliferation, induce angiogenesis, and accelerate invasion, and is responsible for treatment resistance in HCC. Activation of oncogenes or inactivation of tumor suppressors can change signaling pathways and up-regulate HIF-1α expression, leading to HIF-1α activation [23][24]. In the present study, the hepatic expression and circulating levels of HIF-1α in patients with liver disease were investigated in order to elucidate the relationship between HIF-1α level and pathological characteristics, as well as the diagnosis and metastasis of HCC.

Under hypoxic conditions it can be stabilized, binding to specific sites on hypoxia-response target genes, regulating proliferation on a transcriptional level, and activating the expression of many hypoxia-response genes, which are closely involved in energy metabolism, angiogenesis, infiltration, metastasis, and prognosis [25][26]. The positive staining of HIF-1α was brown and granulelike, and mainly present in the cytoplasm, with lower levels in the nucleus. There were obvious differences in HIF- 1α positive expression intensity among different areas of tissues. HIF-1α staining in paracancerous tissues could be seen at significant levels in compressed hepatic cords and central veins [27]. The intensity of HIF-1α expression was significantly higher in paracancerous tissues than in HCC tissues, mainly due to higher levels of necrosis in the latter, representing that there is a very close relationship between high intensity of HIF-1α expression and active proliferation or a hypoxic microenvironment in paracancerous tissues [28].

HCC is mostly characterized by uncontrolled growth of tumor cells. Increasing oxygen consumption results in a hypoxic microenvironment. HIF-1α expression is significantly high in adjacent areas of necrosis and tumor infiltration. Many factors, such as hypoxia, oncogene activation, inactivation of tumor suppressors, growth factors, and inflammatory factors, can up-regulate HIF-1α expression, either directly or indirectly, promoting the transcription of more than 2% of human genes, which are all related to oxygen and energy metabolism [28][29]. Productive nucleic acid metabolism, abnormal gene expression, and development of HCC are closely associated with the state of the surrounding vessels and hypoxic conditions. The level of total RNA was obviously higher in paracancerous tissues than in HCC, indicating that HIF-1α mRNA is involved in cell proliferation, neovascularization, and metastasis and could be a prime target for gene therapy [30][31].

Clinical pathological features of HIF-1α expression indicated that HIF-1α expression intensity and positivity rate were lower in HCC than in paracancerous tissues, which was in accordance with total RNA [32]. HIF-1α positivity rate was associated with tumor diameter, because they were usually single, enveloped, and well-differentiated, and there were more diplonts and less heteromorphism when tumors were small. When tumors increase in size, the biological characteristics changed to the contrary, invasion is therefore strengthened, and tumor blood supply cannot satisfy growth demand [33][34]. HBx and HIF- 1α are both present in the cytoplasm in HCC. Moreover, HBx can up-regulate HIF-1α under normoxic or hypoxic conditions, reinforce HIF-1α transcriptional activity via the MAPK pathway, increase HIF-1α protein levels, induce neovascularization, and thus contribute to metastasis [32]. In the present study, no correlation was found between HIF-1α and HBsAg positivity in HCC and further studies are required to determine whether HIF-1α is associated with HBV.

The prognosis for HCC is poor, and early detection is of the utmost importance. Treatment options are severely limited by the frequent presence of metastases. Although the mechanisms of hepatocarcinogenesis have not been elucidated, a long-lasting inflammation induced by hepatitis virus infection is a definite risk for neoplastic degeneration and the accumulation of genetic alterations [35][36]. Serum AFP is a useful serological marker for monitoring HCC development; however, a high false negative rate has been found when using AFP level alone for monitoring small HCCs [37]. Fragments of circulating HIF-1α could be detected in all patients with HCC with extrahepatic metastasis; as with circulating IGF-II, these results argue for growth factor-dependent HCC development and could provide novel markers of severity and prognosis for HCC. The present data indicate that expression of serum HIF-1α, Ang-2, and VEGF can only be detected in the peripheral blood of patients with HCC. The frequency of circulating HIF-1α and its diagnostic value increases with distal metastases of HCC hepatocytes [38]. The pathological characteristics of serum HIF-1α were associated with the levels of circulating VEGF and Ang-2, the size of the tumor, and extrahepatic metastasis, and but not with patients’ gender, age, and AFP level.

In conclusion, hepatic HIF-1α expression is associated with the development and prognosis of HCC, and circulating HIF-1α level is a useful molecular marker in HCC diagnosis, and for monitoring prognosis. HIF-1α expression in hepatic tissues plays an important role in the development and prognosis of HCC. HIF-1α, as an initial hypoxia moderator, should be a promising molecular target for the development of anti-HCC agents [39]. The intensity of HIF-1α expression was significantly higher in paracancerous tissues than in HCC, mainly due to higher necrosis in the latter, representing the likelihood that there is a very close relationship between a high intensity of HIF-1α expression and active metabolism or hypoxic microenvironment in paracancerous tissues; HIF-1α could therefore be a useful molecular target for gene therapy.

## References

[1] Tang ZY (2002). Small hepatocellular carcinoma: current status and prospects. Hepatobiliary Pancreat Dis Int.

[2] Raza SA, Clifford GM, Franceschi S (2007). Worldwide variation in the relative importance of hepatitis B and hepatitis C viruses in hepatocellular carcinoma: a systematic review. Br J Cancer.

[3] Hui KM (2009). Human hepatocellular carcinoma: expression profiles-based molecular interpretations and clinical applications. Cancer Lett.

[4] Feo F, Frau M, Tomasi ML, Brozzetti S, Pascale RM (2009). Genetic and epigenetic control of molecular alterations in hepatocellular carcinoma. Exp Biol Med (Maywood).

[5] Liu LP, Liang HF, Chen XP, Zhang WG, Yang SL, Xu T, Ren L (2010). The role of NF-kappaB in Hepatitis b virus X protein-mediated upregulation of VEGF and MMPs. Cancer Invest.

[6] Dong ZZ, Yao DF, Wu W, Yao M, Yu HB, Shen JJ, Qiu LW, Yao NH, Sai WL, Yang JL (2010). Delayed hepatocarcinogenesis through antiangiogenic intervention in the nuclear factor-kappa B activation pathway in rats. Hepatobiliary Pancreat Dis Int.

[7] Yao DF, Dong ZZ, Yao M (2007). Specific molecular markers in hepatocellular carcinoma. Hepatobiliary Pancreat Dis Int.

[8] Xie H, Song J, Liu K, Ji H, Shen H, Hu S, Yang G, Du Y, Zou X, Jin H, Yan L, Liu J, Fan D (2008). The expression of hypoxia-inducible factor-1alpha in hepatitis B virus-related hepatocellular carcinoma: correlation with patients' prognosis and hepatitis B virus X protein. Dig Dis Sci.

[9] Daskalow K, Pfander D, Weichert W, Rohwer N, Thelen A, Neuhaus P, Jonas S, Wiedenmann B, Benckert C, Cramer T (2009). Distinct temporospatial expression patterns of glycolysis-related proteins in human hepatocellular carcinoma. Histochem Cell Biol.

[10] Mabjeesh NJ, Amir S (2007). Hypoxia-inducible factor (HIF) in human tumorigenesis. Histol Histopathol.

[11] Patiar S, Harris AL (2006). Role of hypoxia-inducible factor-1alpha as a cancer therapy target. Endocr Relat Cancer.

[12] Weidemann A, Johnson RS (2008). Biology of HIF-1alpha. Cell Death Differ.

[13] Liao D, Johnson RS (2007). Hypoxia: a key regulator of angiogenesis in cancer. Cancer Metastasis Rev.

[14] Copple BL, Bustamante JJ, Welch TP, Kim ND, Moon JO (2009). Hypoxia-inducible factor-dependent production of profibrotic mediators by hypoxic hepatocytes. Liver Int.

[15] Semenza GL (2009). Regulation of oxygen homeostasis by hypoxia-inducible factor 1. Physiology (Bethesda)..

[16] Brahimi-Horn C, Mazure N, Pouyssegur J (2005). Signalling via the hypoxia-inducible factor-1alpha requires multiple posttranslational modifications. Cell Signal.

[17] Yao DF, Jiang H, Yao M, Li YM, Gu WJ, Shen YC, Qiu LW, Wu W, Wu XH, Sai WL (2009). Quantitative analysis of hepatic hypoxia-inducible factor-1alpha and its abnormal gene expression during the formation of hepatocellular carcinoma.. Hepatobiliary Pancreat Dis Int.

[18] Professional Committee of Chinese Anti Cancer Association of liver cancer. (2001). [Clinical diagnostic/staging criteria of primary liver cancer].. Zhonghua Gan Zang Bing Za Zhi.

[19] The Group of Viral Hepatitis Research. (2000). [The prevention and cure scheme of viral hepatitis].. Zhonghua Ganzang Bing Zazhi.

[20] Qian J, Yao D, Dong Z, Wu W, Qiu L, Yao N, Li S, Bian Y, Wang Z, Shi G (2010). Characteristics of hepatic igf-ii expression and monitored levels of circulating igf-ii mRNA in metastasis of hepatocellular carcinoma. Am J Clin Pathol.

[21] (2000). Abnormal expression of hepatoma specific gamma-glutamyl transferase and alteration of gamma-glutamyl transferase gene methylation status in patients with hepatocellular carcinoma. Cancer.

[22] Yoo YG, Kong G, Lee MO (2006). Metastasis-associated protein 1 enhances stability of hypoxia-inducible factor-1alpha protein by recruiting histone deacetylase 1. EMBO J.

[23] Ripoli M, D'Aprile A, Quarato G, Sarasin-Filipowicz M, Gouttenoire J, Scrima R, Cela O, Boffoli D, Heim MH, Moradpour D, Capitanio N, Piccoli C (2010). Hepatitis C virus-linked mitochondrial dysfunction promotes hypoxia-inducible factor 1 alpha-mediated glycolytic adaptation. J Virol.

[24] Cummins EP, Berra E, Comerford KM, Ginouves A, Fitzgerald KT, Seeballuck F, Godson C, Nielsen JE, Moynagh P, Pouyssegur J, Taylor CT (2006). Prolyl hydroxylase-1 negatively regulates IkappaB kinase-beta, giving insight into hypoxia-induced NFkappaB activity. Proc Natl Acad Sci U S A.

[25] Walmsley SR, Print C, Farahi N, Peyssonnaux C, Johnson RS, Cramer T, Sobolewski A, Condliffe AM, Cowburn AS, Johnson N, Chilvers ER (2005). Hypoxia-induced neutrophil survival is mediated by HIF-1alpha-dependent NF-kappaB activity. J Exp Med.

[26] Shin DH, Li SH, Yang SW, Lee BL, Lee MK, Park JW (2009). Inhibitor of nuclear factor-kappaB alpha derepresses hypoxia-inducible factor-1 during moderate hypoxia by sequestering factor inhibiting hypoxia-inducible factor from hypoxia-inducible factor 1alpha. FEBS J.

[27] van Uden P, Kenneth NS, Rocha S (2008). Regulation of hypoxia-inducible factor-1alpha by NF-kappaB. Biochem J.

[28] Büchler P, Reber HA, Tomlinson JS, Hankinson O, Kallifatidis G, Friess H, Herr I, Hines OJ (2009). Transcriptional regulation of urokinase-type plasminogen activator receptor by hypoxia-inducible factor 1 is crucial for invasion of pancreatic and liver cancer. Neoplasia.

[29] Hamaguchi T, Iizuka N, Tsunedomi R, Hamamoto Y, Miyamoto T, Iida M, Tokuhisa Y, Sakamoto K, Takashima M, Tamesa T, Oka M (2008). Glycolysis module activated by hypoxia-inducible factor 1alpha is related to the aggressive phenotype of hepatocellular carcinoma. Int J Oncol.

[30] Forooghian F, Das B (2007). Anti-angiogenic effects of ribonucleic acid interference targeting vascular endothelial growth factor and hypoxia-inducible factor-1alpha. Am J Ophthalmol.

[31] Nasimuzzaman M, Waris G, Mikolon D, Stupack DG, Siddiqui A (2007). Hepatitis C virus stabilizes hypoxia-inducible factor 1alpha and stimulates the synthesis of vascular endothelial growth factor. J Virol.

[32] Dong ZZ, Yao DF, Yao M, Qiu LW, Zong L, Wu W, Wu XH, Yao DB, Meng XY (2008). Clinical impact of plasma TGF-beta1 and circulating TGF-beta1 mRNA in diagnosis of hepatocellular carcinoma. Hepatobiliary Pancreat Dis Int.

[33] Lee TK, Poon RT, Yuen AP, Ling MT, Wang XH, Wong YC, Guan XY, Man K, Tang ZY, Fan ST (2006). Regulation of angiogenesis by Id-1 through hypoxia-inducible factor-1alpha-mediated vascular endothelial growth factor up-regulation in hepatocellular carcinoma. Clin Cancer Res.

[34] Zhu H, Chen XP, Luo SF, Guan J, Zhang WG, Zhang BX (2005). Involvement of hypoxia-inducible factor-1-alpha in multidrug resistance induced by hypoxia in HepG2 cells. J Exp Clin Cancer Res.

[35] Wada H, Nagano H, Yamamoto H, Yang Y, Kondo M, Ota H, Nakamura M, Yoshioka S, Kato H, Damdinsuren B, Tang D, Marubashi S, Miyamoto A, Takeda Y, Umeshita K, Nakamori S, Sakon M, Dono K, Wakasa K, Monden M (2006). Expression pattern of angiogenic factors and prognosis after hepatic resection in hepatocellular carcinoma: importance of angiopoietin-2 and hypoxia-induced factor-1 alpha. Liver Int.

[36] Yao DF, Wu XH, Zhu Y, Shi GS, Dong ZZ, Yao DB, Wu W, Qiu LW, Meng XY (2005). Quantitative analysis of vascular endothelial growth factor, microvascular density and their clinicopathologic features in human hepatocellular carcinoma. Hepatobiliary Pancreat Dis Int.

[37] Wu W, Yao DF, Yuan YM, Fan JW, Lu XF, Li XH, Qiu LW, Zong L, Wu XH (2006). Combined serum hepatoma-specific alpha-fetoprotein and circulating alpha-fetoprotein-mRNA in diagnosis of hepatocellular carcinoma. Hepatobiliary Pancreat Dis Int.

[38] Kulshreshtha R, Davuluri RV, Calin GA, Ivan M (2008). A microRNA component of the hypoxic response. Cell Death Differ.

[39] Takahashi Y, Nishikawa M, Takakura Y (2008). Inhibition of tumor cell growth in the liver by RNA interference-mediated suppression of HIF-1alpha expression in tumor cells and hepatocytes. Gene Ther.

